# Increased Workload for Systematic Review Literature Searches of Diagnostic Tests Compared With Treatments: Challenges and Opportunities

**DOI:** 10.2196/medinform.3037

**Published:** 2014-05-27

**Authors:** Henry Petersen, Josiah Poon, Simon K Poon, Clement Loy

**Affiliations:** ^1^School of Information TechnologiesFaculty of Engineering and ITUniversity of SydneySydneyAustralia; ^2^School of Public HealthFaculty of MedicineUniversity of SydneySydneyAustralia

**Keywords:** meta-analysis, data mining, review literature, information storage and retrieval, classification and clustering

## Abstract

**Background:**

Comprehensive literature searches are conducted over multiple medical databases in order to meet stringent quality standards for systematic reviews. These searches are often very laborious, with authors often manually screening thousands of articles. Information retrieval (IR) techniques have proven increasingly effective in improving the efficiency of this process. IR challenges for systematic reviews involve building classifiers using training data with very high class-imbalance, and meeting the requirement for near perfect recall on relevant studies. Traditionally, most systematic reviews have focused on questions relating to treatment. The last decade has seen a large increase in the number of systematic reviews of diagnostic test accuracy (DTA).

**Objective:**

We aim to demonstrate that DTA reviews comprise an especially challenging subclass of systematic reviews with respect to the workload required for literature screening. We identify specific challenges for the application of IR to literature screening for DTA reviews, and identify potential directions for future research.

**Methods:**

We hypothesize that IR for DTA reviews face three additional challenges, compared to systematic reviews of treatments. These include an increased class-imbalance, a broader definition of the target class, and relative inadequacy of available metadata (ie, medical subject headings (MeSH) terms for medical literature analysis and retrieval system online). Assuming these hypotheses to be true, we identify five manifestations when we compare literature searches of DTA versus treatment. These manifestations include: an increase in the average number of articles screened, and increase in the average number of full-text articles obtained, a decrease in the number of included studies as a percentage of full-text articles screened, a decrease in the number of included studies as a percentage of all articles screened, and a decrease in the number of full-text articles obtained as a percentage of all articles screened. As of July 12 2013, 13 published Cochrane DTA reviews were available and all were included. For each DTA review, we randomly selected 15 treatment reviews published by the corresponding Cochrane Review Group (N=195). We then statistically tested differences in these five hypotheses, for the DTA versus treatment reviews.

**Results:**

Despite low statistical power caused by the small sample size for DTA reviews, strong (*P*<.01) or very strong (*P*<.001) evidence was obtained to support three of the five expected manifestations, with evidence for at least one manifestation of each hypothesis. The observed difference in effect sizes are substantial, demonstrating the practical difference in reviewer workload.

**Conclusions:**

Reviewer workload (volume of citations screened) when screening literature for systematic reviews of DTA is especially high. This corresponds to greater rates of class-imbalance when training classifiers for automating literature screening for DTA reviews. Addressing concerns such as lower quality metadata and effectively modelling the broader target class could help to alleviate such challenges, providing possible directions for future research.

## Introduction

### Background

Systematic reviews are a key component in evidence-based medicine and are widely regarded as the highest form of medical evidence [1]. A number of organizations such as the Cochrane collaboration exist to facilitate the generation and dissemination of systematic reviews for a range of clinical questions and fields. For example, Cochrane maintains the Cochrane database of systematic reviews; an extensive database which, at the end of the year 2013 contained over 5000 reviews. Traditionally, systematic reviews have focused on questions related to medical interventions, however recently there has been increasing demand for reviews from other areas (ie, etiology, diagnosis, prognosis, etc). In particular, there has been a substantial increase in demand for reviews of diagnostic test accuracy (DTA) leading to the formation of the Cochrane diagnostic test accuracy working group in 2003.

The high potential cost of omitting relevant studies from medical decision making is well established [2]. In order to meet the stringent recall requirements for systematic reviews, authors must conduct highly sensitive, detailed literature searches. To minimize the possibility of error, these searches in most cases are manually conducted and are eventually time consuming [1]. It is not unusual for an individual review to be conducted over the course of months or even years [3]. As the demand for systematic reviews increases, it is apparent that methods to automate or expedite the review process are essential [4].

In recent times there has been much interest expressed by the information retrieval (IR) community on increasing the automation of literature searches for systematic reviews [5-7]. This automation process typically involves a set of labelled training instances (articles marked as relevant or irrelevant to the target review), and a classification algorithm which is run on these instances to “train” a mapping function (“classifier”) from instances to labels. From the perspective of training such a classifier, systematic reviews present several challenges: the training data is highly imbalanced (ie, the number of included studies will be small as a percentage of all training examples) [5], there is a need for near perfect recall, and it is not clear how to best incorporate partial automation into the systematic review process. Despite the above concerns, these methods have met with limited success. Thus further improvements on the methodology is a clear mandate [8,9].

While the medical community has noted a number of challenges facing authors of DTA reviews [10], there has been no analysis on the differences between reviews of DTA and interventions as an IR problem. For the purposes of this study we consider the term “diagnostic test accuracy” to be defined as broadly as possible (we do not limit ourselves to any particular field or study design and consider a DTA review to be any review evaluating the accuracy of a specific diagnostic test). From an IR perspective one of the key challenges in retrieval for systematic reviews is the level of class-imbalance. We identify DTA reviews as a subclass with particularly high class-imbalance rates through a statistical analysis of the reported literature searches from a number of Cochrane reviews of DTA and treatment. Our analysis also identifies two potential causes, which from an IR standpoint provide potential starting points in reducing the additional level of class-imbalance.

The remainder of this section briefly describes the literature search process for systematic reviews and previous applications of IR to the systematic review process. For the sake of brevity, only prior work relating to IR challenges from literature searches where differences between DTA and interventions exist are covered. The interested reader is directed to other literature for more information [11,12].

### Overview of Systematic Reviews

While the exact process for conducting a systematic review varies according to the type of clinical question (ie, diagnosis, intervention, etiology), all systematic reviews can be said to follow several steps [13]. These include question and inclusion criteria formulation, literature search, literature screening, quality assessment, and data synthesis, analysis and interpretation.

For brevity’s sake a summary of the entire systematic review process is not presented. Instead we include a brief summary of the first three stages. For further information the interested reader is directed to literature such as Wright et al [1] or the Cochrane handbooks for reviews of interventions [14] and DTA [15].

### Question and Inclusion Criteria Formulation

Systematic reviews begin with the formulation of a highly specific research question and associated inclusion criteria. Inclusion criteria for Cochrane Systematic Reviews are formulated according to specific concepts that depend on the type of clinical question being answered. For example, in Cochrane Reviews of diagnostic test accuracy, separate criteria are formulated for the type of study, index and comparator tests, target condition and the desired reference standard [15]. A similar set of criteria (referred to as the PICO criteria—Population, Intervention, Comparison, Outcome) exists for questions related to interventions [14].

### Literature Search

Review authors will then query multiple databases to identify potential relevant studies (usually medical literature analysis and retrieval system online (MEDLINE) and excerpta medica database (EMBASE) at a minimum although other resources do exist). In order to facilitate this process, citations indexed in these databases are typically annotated with entries from a controlled hierarchy of medical concepts that can be used for search and retrieval (examples include the MeSH for MEDLINE or EMTREE for EMBASE).

Literature searches for Cochrane Systematic Reviews are typically conducted by identifying references containing relevant MeSH and free text terms. Cochrane Reviews of interventions usually identify multiple MeSH terms relating to several key concepts of the review. Searches for each of these concepts are run using the identified MeSH terms, with the union of the search results selected for further screening. Literature searches for systematic reviews of diagnostic test accuracy are similar, however the methodological search filter is often omitted [16]. While much research has been done on developing highly sensitive DTA filters [17-22], the broader community has yet to develop a consensus on their use in DTA reviews (for example the Cochrane handbook for DTA reviews recommends against the "routine use of methodology search filters"[15]).

### Literature Screening

References returned by the literature search are manually compared against the inclusion criteria for the reviews in a two stage process. Initially, two reviewers independently screen title and abstracts for all references, with full-text articles obtained for any potentially relevant citations. These full-text articles are then screened again by both reviewers.

To meet the requirement for near perfect recall, the number of references screened can often be many times greater, often one or two orders of magnitude than the number included in the final review. Karimi et al noted that when screening citations, each individual document may require several minutes to process [23]. It is apparent that even small reductions in the number of citations screened could result in a significant reduction in reviewer time and effort. Still, the high rates of class-imbalance, combined with the stringent recall requirements present a significant IR challenge.

### State of the Art

A major concern for IR with systematic reviews is dealing with highly imbalanced training data when building classifiers (ie, the number of available examples of relevant articles for a given review will be small relative to the number of irrelevant ones, leading to models which can be biased towards the non-relevant studies). Addressing this class-imbalance has been a key feature of much of the relevant IR literature [5]. Existing techniques have met with some success, however improvements in performance are still required, especially for those with higher rates of imbalance [8,9].

In addition to high levels of class-imbalance, IR for systematic reviews must also meet stringent recall requirements. In other words, there is a large difference in the cost of false positive and false negative errors for IR algorithms when identifying citations for inclusion into systematic reviews. Prior work addressing this issue include the modified voting perceptron method of Cohen et al [24], the factorized Complement Naïve Bayes model of Matwin et al [25], and support vector machine based approaches by Cohen et al [6,26] and Wallace et al [7,27]

Attention has also been directed towards the best approaches on combining IR techniques with the systematic review process. Frunza et al [5] describe an approach based on having authors manually screen some percentage of all citations, which are then used as training data to build a classifier to be run on the remaining articles. In contrast, Wallace et al [7, 27, 28] describe an active learning approach, where the classifier is built in an iterative process. Here the algorithm particularly selects those citations for which manual annotation would provide the greatest improvement. Finally, work exists addressing the similar task of identifying studies to update existing reviews [24,29]. Automation of the review update task is similar to classification for the initial review, however it fits much better with the traditional classification model in which separate training and test sets are used (ie, annotations from the original search can be used to train the classifier for the update task).

There has also been some interest in applying classification to assign relevant MeSH terms to citations from MEDLINE [30], as well as retrieval of studies of high methodological quality [31]. For example, on employing articles retrieved from the American College of Physicians (ACP) journal club as training data, Aphinyanaphongs et al [31] evaluated a range of common algorithms and reported that their preliminary results showed good performance on identifying high quality DTA studies.

While such results may at first seem to contradict the difficulty of creating high quality DTA classifiers, the distinction between general retrieval of DTA studies and retrieval of 'high quality' DTA studies should be noted. Aphinyanaphongs et al trained their classifier based on citations retrieved from the ACP journal clubs meta-publication which applies strict quality criteria to determine if a citation should be included [31]. As the ACP restrict inclusion to high quality articles this could be expected to significantly reduce variance across the target class, reducing the complexity of the task for any prospective classifier.

## Methods

### Overview

This section outlines three hypotheses regarding technical challenges faced by both authors and IR researches for DTA reviews. These hypotheses relate to differences in the literature search process between systematic reviews of DTA and treatment. Hypothesis A relates to the screening process as a whole, while Hypotheses B and C relate to stage 2 and stage 1 screening respectively. We describe one or more expected manifestations for each hypothesis. The analysis in this paper reports whether or not the expected manifestations can be observed and if the observations are statistically significant. A tabular summary linking each hypothesis, manifestation, and screening stage is presented in Table 1.

### Hypothesis A: Increased Workload for DTA Reviews

A major practical issue when conducting systematic reviews is the workload generated by the volume of citations needing to be screened. Most IR research for systematic reviews has focused specifically on how to deal with the very high rates of class-imbalance caused by this volume of data. Substantial progress has been made, however it can by no means be considered a solved problem.

This article claims that the number of citations to be screened at each stage of the literature search process is higher for DTA reviews than for those of the treatment. From an IR perspective, this increases the already large class-imbalance between the number of included and excluded studies, thereby again increasing the difficulty of what was already very challenging. Assuming this to be true, one could then expect the following manifestations (restated in Table 1): First, the mean number of search results to be screened will be higher for DTA reviews than for those of treatment. Second, the mean number of full-text articles to be screened will be higher for DTA reviews than for those of treatment. Finally, the number of included studies as a percentage of the number of full-text articles screened would be lower for DTA reviews than for treatment.

### Hypothesis B: Increased Target Class Heterogeneity for DTA

The relative heterogeneity of what exactly constitutes a DTA study can be problematic when screening literature for DTA reviews. Quoting from Whiting et al [19], diagnostic test accuracy studies “are heterogeneous, exploring a range of diagnostic techniques and strategies, and are likely to have been conducted using a variety of methods”. In addition, there are examples (such as some cohort studies) where one could derive sensitivity and specificity despite the authors not having explicitly calculated them. The ideal DTA filter should be highly sensitive and would include studies such as these.

Our paper suggests that due to this increased difficulty, the percentage of irrelevant citations that cannot be identified on title and abstract alone will be larger for DTA reviews than for treatment. Assuming this to be true, we can expect the following manifestations (restated in Table 1): The mean number of full-text articles to be screened will be higher for DTA reviews than for those of treatment, and the number of included studies as a percentage of the number of full-text articles screened would be lower for DTA reviews than for treatment.

Intuitively, if a given study type is more challenging to identify than another, it can be expected that an author would need to expend greater effort on discerning similar studies. This increased effort could take the form of additional time to screen individual citations, or screening more citations in greater detail (ie, examining the full-text article). Due to the high cost of false negative classifications, it is reasonable to assume that any ambiguity in the initial screening stage would be resolved by obtaining the full-text article rather than putting more effort on the title and abstract. As such, assuming DTA studies to be inherently more challenging to identify than randomized controlled trials, we would expect to observe more full-text articles being screened when conducting DTA reviews.

### Hypothesis C: Decreased Suitability of Metadata for DTA

Appropriate use of high quality metadata (ie, MeSH terms for MEDLINE) in literature searches is crucial to generate a manageable number of citations while still remaining confident that no relevant ones would be omitted. It is common to identify thousands of citations at this stage. It follows that as the quality of the available metadata decreases, the total number of citations one would need to screen to maintain this confidence would increase.

It has been noted within the literature that the metadata in many medical databases are more suited to describing concepts related to treatment as opposed to diagnosis [15]. For example, while high quality MeSH terms exist for study types such as randomized controlled trials, the same cannot be said for studies of diagnostic test accuracy. From Whiting et al [19]: “Although MEDLINE includes a number of medical subject headings (MeSH) that capture outcome measures used in test accuracy studies (eg, sensitivity and specificity), these terms are not specific to test accuracy studies and are inconsistently applied by indexers”.

This article claims that the quality of metadata is typically lower for DTA reviews than for treatment. Therefore we can expect the following manifestations in literature searches for systematic reviews (restated in Table 1): The mean number of search results to be screened will be higher for DTA reviews than for those of treatment, and the number of full-text articles retrieved as a percentage of the total search results would be lower for DTA reviews than for treatment.

### Data Collection

We have identified five expected manifestations of the stated hypotheses on the literature searches for DTA reviews (restated in Table 1). In order to test these claims, summaries of the literature search and screening stages were extracted from a sample of DTA and treatment reviews. Data collected included the number of references retrieved by the original search (SR), the number of references for which full-text papers were screened (FT), the number of references included in the final meta-analysis (INC), and the paired ratios between each of the collected statistics.

**Table 1:** List of expected manifestations (differences between DTA and treatment reviews) for all hypotheses.

Manifestation	Description	Hypothesis A: Increased Workload	Hypothesis B: Increased Target Class Heterogeneity	Hypothesis C: Decreased Suitability of Metadata
FT^a^	The mean number of full-text articles screened would be higher for DTA reviews than for treatment	Yes	-	-
SR^b^	The mean number of search results would be higher for DTA reviews than for treatment	Yes	-	Yes
INC^c^/ FT	The number of included studies as a percentage of the number of full-text articles screened would be lower for DTA reviews than for treatment	-	Yes	-
INC/SR	The number of included studies as a percentage of the total number of search results would be lower for DTA reviews than for treatment	Yes	-	-
FT/SR	The number of full-text articles retrieved as a percentage of the total search results would be lower for DTA reviews than for treatment	-	-	Yes

^a^number of references for which full-text papers were screened

^b^number of references retrieved by the original search

^c^number of references included in the final meta-analysis

Systematic reviews can be conducted and reported according to varying standards of rigor. This could be problematic for the purposes of our evaluation, as ideally the variation between two samples should be restricted to one review type (ie, DTA or treatment). For systematic reviews published by the Cochrane collaboration, authors are required to follow strict guidelines outlined in the Cochrane handbooks for treatment and DTA reviews [14,15]. Reviews published by Cochrane are widely regarded as meeting very high procedural and reporting standards, and their published guidelines for reviews of DTA and treatment contain a number of shared protocols. As we wish to restrict differences between the samples to whether the reviews are of treatment or DTA, the analysis reported in this paper is performed exclusively on a subset of the Cochrane database.

As of the search date (July 12 2013), Cochrane had published 13 complete systematic reviews of DTA (one from each of the acute respiratory infections [ARI], airways, back, bone, joint, and muscle trauma [BJMT], eyes and vision, gynecological cancer, pregnancy, renal, and stroke Cochrane review groups [CRG], two from the infectious diseases CRG, and three from the Back CRG). A copy of each DTA review was obtained. For each DTA review, 15 non-withdrawn treatment reviews were selected at random from those published by the corresponding CRG. Stratifying the data in this way was intended to account for any variation in search procedures across CRGs, as well as the availability of data within each field generally. A summary of the number of selected treatment reviews for each CRG is presented in Table 2. A list of each selected diagnostic and treatment review is included in the Multimedia Appendix 1. One author then manually collected the desired statistics from the values reported in the literature search summary from each review.

It is important to recall that depending on the specific conditions of each review (DTA or treatment) changes in the search process may be made to find the desired balance between search sensitivity and reviewer workload. Using the values reported by the reviewers (as opposed to manually re-running searches, possibly with the inclusion of more or less sensitive filters) had the added benefit of taking into account the review authors conclusions for the specific domain of each review.

**Table 2:** Summary of the total number of DTA and treatment reviews randomly selected for inclusion in our analysis, ordered by CRG.

Cochrane review groups	DTA reviews	Treatment reviews
Acute respiratory infections	1	15
Airways	1	15
Back	3	45
Bone, joint, and muscle trauma	1	15
Eyes and vision	1	15
Gynecological cancer	1	15
Infectious diseases	2	30
Pregnancy	1	15
Renal	1	15
Stroke	1	15
Total	13	195

Not all reviews reported the number of citations obtained at each stage of the literature search (eg, some would report only the number of included and full-text articles). Where values were missing or unclear, we made an attempt to contact the review authors by email. If no data could be obtained, a blank value was recorded and the review would be omitted from analyses involving the missing statistical data. For computational reasons, extracted values equal to 0 were also omitted. A summary of the number of extracted values for all data types is given in Table 3. For example, of the 195 randomly selected treatment reviews, the number of full-text articles examined could not be obtained from 62 reviews, hence the number of collected data points for the number of full-text articles in treatment reviews is 133 (as reported in row 2 of Table 3).

**Table 3:** Table 3. Summary of the sample sizes (number of reviews reporting nonzero values) for evaluating each of the expected manifestations.

	DTA	Treatment
DATA_INC_	13/13	186/195
DATA_FT_	12/13	133/195
DATA_SR_	13/13	101/195
DATA_INC / FT_	12/13	126/195
DATA_INC / SR_	13/13	95/195
DATA_FT / SR_	12/13	92/195

### Analysis

Based on prior experience, we expected that the number of reported studies for the literature searches would be heavily skewed. This expectation is supported by comparing the mean and median values for each statistics from the collected treatment reviews (see Table 4); for 5 out of the 6 statistics the mean is approximately twice the value of the median. For example, the number of reported search results collected includes a number of values describing unusually large literature searches. Such values significantly affect the skewedness of the collected data, substantially increasing the mean without affecting the median.

**Table 4:** Ratio between mean and median for collected treatment reviews.

	Mean	Median	Mean / Median
DATA_INC_	19.56	11.0	1.78
DATA_FT_	71.89	33.00	2.18
DATA_SR_	1799.04	900.00	2.00
DATA_INC / FT_	0.394	0.357	1.11
DATA_INC / SR_	0.033	0.013	2.47
DATA_FT / SR_	0.099	0.046	2.13

In order to compensate for the level of skewness, all reported statistical comparisons are performed using an unequal variance *t* test on ranked data (ie, as an approximation to a non-parametric test); each individual data point is replaced by its index in the sorted set of data. If multiple data points shared a common value the ranked values were averaged. Summaries of the unranked and ranked data are presented in Table 5 and Table 6.

To further illustrate the ranking process, the mean number of search results obtained (as reported in Table 5) was 5144.23 for DTA reviews and 1799.04 for treatment reviews. When the 13 DTA and 101 treatment data points were combined and sorted however, the mean position for DTA reviews was 85.54 and that for the treatment reviews was 52.76 (as reported in Table 6).

**Table 5:** Summary of mean values for collected statistics.

	Mean_DTA_	Mean_Treat_	Mean_DTA_/ Mean_Treat_
DATA_FT_	191.92 (n=13,s=233.51)	71.89 (n=133,s=154.76)	2.67
DATA_SR_	5144.23 (n=13,s=4109.78)	1799.04 (n=101,s=2530.11)	2.86
DATA_INC / FT_	0.191 (n=13,s=0.11)	0.394 (n=126,s=0.24)	0.49
DATA_INC / SR_	0.021 (n=13,s=0.036)	0.033 (n=95,s=0.049)	0.63
DATA_FT / SR_	0.087 (n=13,s=0.124)	0.100 (n=92,s=0.156)	0.87

**Table 6:** Summary of ranked data for collected statistics.

	Mean_DTA_	Median_DTA_	Mean_Treat_	Median_Treat_
DATA_FT_	110.67 (n=12,s=27.64)	113.0	68.51 (n=133,s=41.16)	67.0
DATA_SR_	85.54 (n=13,s=27.84)	94.0	52.76 (n=101,s=31.62)	52.0
DATA_INC / FT_	35.67 (n=12,s=24.69)	29.0	71.63 (n=126,s=39.60)	73.5
DATA_INC / SR_	40.54 (n=13,s=31.12)	35.0	55.27 (n=95,s=30.76)	56.0
DATA_FT / SR_	47.5 (n=12,s=30.18)	45.5	52.02 (n=92,s=29.97)	53.5

## Results

### Overview

The results section is divided into one section for each of the three proposed hypotheses. Summaries of each hypothesis, along with the expected and observed manifestations is presented in Table 7.

**Table 7:** Summary linking each hypothesis, expected manifestation, and literature screening stage.

	Hypothesis A: Increased workload	Hypothesis B: Increased target class heterogeneity	Hypothesis C: Decreased suitability of metadata
Total articles screened	Increase	-	Increase
	5144.2 _DTA_ > 1799.0_TR_ (*P*=.002)		5144.2 _DTA_ > 1799.0_TR_ (*P*=.002)
Full-text articles obtained	Increase	-	Decreased as a % of total articles screened
	191.9_DTA_> 71.9_TR_ (*P*<.001)		0.087_DTA_< 0.100_TR_ (*P*=.65)
Included Articles	Decrease as a % of total articles screened	Decreased as a % of full-text articles obtained	-
	0.021_DTA_< 0.033_TR_ (*P*=.14)	0.191_DTA_< 0.394_TR_ (*P*<.001)	

### Hypothesis A: Increased Workload for DTA Reviews

Comparing the mean absolute number of the search results obtained we observe a 186% increase for reviews of DTA when compared to reviews of interventions (5144.2 vs 1799.0). There was strong evidence that this difference was statistically significant (unequal variance *t* test on ranked data, *P*=.002). Similarly for the mean number of full-text articles obtained we can observe an increase of 167% (191.9 vs 71.9). Again, there was very strong evidence that this difference was statistically significant (unequal variance *t* test on ranked data, *P*<.001).

We note not only the statistically significant difference in means, but also the substantial difference in effect size. The magnitude of the difference supports the claim that identification of relevant papers is noticeably more complicated for DTA reviews than for those of treatment, and also that there is an increase in difficulty both for authors and any prospective IR system.

Considering the number of included studies as a proportion of the total search results, a decrease of approximately 35% is observed for DTA reviews when compared to reviews of treatment (0.021 vs 0.033). However, despite the large magnitude of the difference there is insufficient evidence to claim statistically significance (unequal variance *t* test on ranked data, *P*=.14). However, the authors urge caution in concluding that no difference exists (see discussion).

### Hypothesis B: Increased Target Class Heterogeneity for DTA

Comparing the number of included studies as a percentage of full-text articles examined, an increase of approximately 106% is observed for DTA reviews when compared to those for treatment (0.191 vs 0.394). Very strong evidence was obtained that this difference was significant (unequal variance *t* test on ranked data, *P*<.001).

Again, we note the substantial difference in the observed effect size here. Its magnitude indicates the increased practical difficulty of screening a potentially relevant article for inclusion in a DTA review.

### Hypothesis C: Decreased Suitability of Metadata for DTA

As stated in the results section for Hypothesis A, strong evidence was obtained to support an increase in the mean absolute number of search results obtained when comparing reviews of DTA and treatment (unequal variance *t* test on ranked data, *P*=.002). When looking at the number of full-text articles retrieved as a percentage of total search results, one can observe a decrease of approximately 13% for DTA reviews when compared to treatment reviews (0.087 vs 0.100). However, there is insufficient evidence to identify a statistically significant difference (unequal variance *t* test on ranked data, *P*=.65). As with the observed mean number of included studies as a percentage of search results, the authors urge caution in concluding that no difference exists, and discuss possible reasons in the following section.

## Discussion

### Principal Findings

As observed from the reported *P* values in Table 7, there is very strong evidence that the number of articles at each stage of the screening process is higher for DTA reviews than for those of treatment, in support of hypothesis A (and hypothesis C in the case of an increased number of raw search results). This demonstrates a significant increase in the required workload for systematic reviews of diagnostic test accuracy. In addition, very strong evidence is obtained in support of hypothesis B. However, the p-values obtained for both the number of included and full-text articles retrieved as a percentage of the total search results were insufficient to ascertain a statistically significant difference between the means for DTA and treatment reviews.

As reported in Table 5 and 6, the standard deviation for all results is quite large. In addition, our analysis is limited in that only 13 completed Cochrane DTA reviews existed as on the search date. This small sample size, combined with the large standard deviations results in relatively low power. There is a possibility that the negative results reported for the included and full-text articles as a percentage of total search results were type II errors. This possibility is enhanced by the relatively large magnitude of the differences in sample means (see Table 5). Of course, it is impossible to say for certain until more data is available.

The authors note that while the analysis does not support the claim of sub-optimal metadata for DTA reviews, such a claim is not new and is supported by previously published literature. In addition to the lack of a definitive MeSH term for DTA studies, the Cochrane Handbook for reviews of DTA studies [15] notes that many index and reference tests employed during DTA studies have no corresponding MeSH term. From the handbook: a “database of names used to describe index tests and reference standards is being built”. However it is not complete as yet and due to the size of databases like MEDLINE and EMBASE, it is unlikely to be able to be applied retrospectively.

The reported results (summarized in Table 7) combined with the substantial difference in observed effect sizes lead the authors to conclude that the analysis supports the claim that DTA reviews present additional IR challenges. The magnitude of the difference in effect sizes is of particular importance as it implies a practical difference in the level of effort required for DTA and treatment reviews. They note the limitations of the study due to the small sample size of available DTA reviews. Further analysis needs to be done when more data is available.

It is interesting to note that the expected manifestations of hypothesis B (increased target class heterogeneity) could be said to drive the expected increase in workload during stage 2 screening described in hypothesis A. Similarly, hypothesis C (sub-optimal metadata) could be said to drive the increased workload in stage 1. This provides an interesting guide to any future work on the application of data mining to DTA reviews; by addressing these challenges the comparative difficulty of DTA reviews can be reduced.

We also like to mention that the hypotheses discussed in this paper could have additional manifestations throughout the review in addition to those in the literature search and screening stages. For example, the increased range of study designs and analysis methodologies (hypothesis B) could lead to increased difficulty in performing or interpreting any subsequent meta-analysis. As the focus of this paper is the literature search/screening stages of DTA reviews (and due to the inability to observe such manifestations in our data) we do not consider such manifestations in our work, however such a study in future may be interesting.

### Strengths and Limitations

To the best of our knowledge, this is the first study directly comparing reviewer workload for literature screening between systematic reviews of DTA and treatment. In addition, as stated in the data collection section, basing the comparison of DTA and treatment samples off the reported number of citations screened (rather than rerunning searches where applicable) is an advantage of our study. Such an approach implicitly takes into account decisions by authors about the required sensitivity of the initial search, which can be expected to differ across individual reviews and clinical domains.

There are several limitations to our study. Firstly, the relatively small number of Cochrane DTA reviews published as of the search date (n=13) results in statistical analysis with low power. As more data is available, future studies that permit comparison of DTA and treatment reviews in fields beyond those published to date by the 10 CRGs, would be of interest. Our results may also be biased towards Cochrane Reviews, as our analysis was performed purely on reviews collected from the Cochrane database of systematic reviews. As discussed earlier in the article, we believe this decision to be justified as it helps restrict the variance between two samples to clinical type (ie, DTA or treatment). Nonetheless this needs to be considered when interpreting the results of our study.

### Conclusions

We demonstrate an increase in practical difficulty when screening literature for DTA reviews as compared to treatment. In addition, some potential causes for this additional difficulty at each stage of the literature search process are presented. We make three main conclusions in this article: first, the overall reviewer workload during literature screening is higher for DTA reviews than for treatment, as evidenced by the larger number of citations obtained at each stage of the literature screening process. Second, the target class of studies included in DTA reviews is broader than the corresponding class for reviews of treatment, as evidenced by the lower number of included studies as a percentage of full-text articles screened. Finally, we provide partial statistical evidence to support claims of the relative unsuitability of available metadata for DTA reviews. We note that future analyses with higher statistical power would be of greater interest.

This article provides a strong case for increased attention from the IR community on systematic reviews of DTA. Such work to address the challenges discussed in this paper could lead to genuine reductions in the workload and difficulty of conducting DTA reviews. One possible direction for future research includes developing high quality classifiers for DTA studies. This could help build consensus with the goal of widespread use of methodological search filters, similar to the current practice for Cochrane Reviews of treatment. As authors for DTA reviews must take into account that relevant data for any meta-analysis can often be synthesized from a range of studies (for example, non-DTA studies reporting individualized patient data [32-35]), this task could be further refined to develop classifiers for things such as individual study designs (ie, cohort study, case-control study), or to simply identify studies that report things like individualized patient data for a given test. Another advantage of individual patient data is that it will allow for a more tailored application to clinical scenarios via subgroup analysis.

In addition, given the size and scope of resources such as MeSH, it is unreasonable to expect all relevant metadata to be assigned to all references. The development of classifiers to assign interesting or relevant MeSH terms would help to increase the recall of interesting terms, potentially allowing for the creation of shorter, more specific queries. Such classifiers could also be used to apply newer MeSH terms retrospectively in existing databases. Finally, a third potential direction includes the application of data mining to identify which MeSH terms have particularly high discriminative power for DTA reviews. This task works in conjunction with the development of MeSH classifiers. Alternatively, data mining could be applied to identify clusters of citations that do not correspond to specific MeSH terms but nonetheless contain good discriminative power.

Over time, as the above concerns are addressed it could be expected that the required workload for DTA and treatment reviews converge. However there are two reasons for which research addressing IR for reviews with very high levels of class-imbalance (such as those currently observed for DTA reviews) is also required: first, the number of references screened for systematic reviews is heavily right-tailed (see data collection). For both treatment and DTA, dealing with reviews at the extreme end of the spectrum is an open problem [9]. And second, while it can be expected that future developments in mitigating the above challenges will reduce the levels of class-imbalance, it is unlikely that an optimal solution will be found in the near future. In addition, while efforts are occasionally made to retrospectively update metadata for databases such as MEDLINE and EMBASE where a sufficient need can be demonstrated (eg, the MeSH re-tagging project for randomized controlled trials [36]), the cost and difficulty of such tasks implies that some challenges are unlikely to be entirely solved.

## Multimedia Appendix 1

List of Cochrane Reviews included in the analysis.

## References

[1] Wright RW, Brand RA, Dunn W, Spindler KP (2007). How to write a systematic review. Clin Orthop Relat Res.

[2] McLellan F (2001). 1966 and all that-when is a literature search done?. Lancet.

[3] Sampson M, Shojania KG, Garritty C, Horsley T, Ocampo M, Moher D (2008). Systematic reviews can be produced and published faster. J Clin Epidemiol.

[4] Tsafnat G, Dunn A, Glasziou P, Coiera E (2013). The automation of systematic reviews. BMJ.

[5] Frunza O, Inkpen D, Matwin S (2010). Building systematic reviews using automatic text classification techniques. Proceedings of the 23rd International Conference on Computational Linguistics: Posters.

[6] Cohen AM, Ambert K, McDonagh M (2009). Cross-topic learning for work prioritization in systematic review creation and update. J Am Med Inform Assoc.

[7] Wallace BC, Small K, Brodley CE, Trikalinos TA (2010). Active learning for biomedical citation screening.

[8] Cohen AM (2011). Performance of support-vector-machine-based classification on 15 systematic review topics evaluated with the WSS@95 measure. J Am Med Inform Assoc.

[9] Matwin S, Kouznetsov A, Inkpen D, Frunza O, O'Blenis P (2010). Performance of SVM and Bayesian classifiers on the systematic review classification task. J Am Med Inform Assoc.

[10] Devillé WL, Buntinx F, Bouter LM, Montori VM, de Vet HC, van der Windt DA, Bezemer PD (2002). Conducting systematic reviews of diagnostic studies: didactic guidelines. BMC Med Res Methodol.

[11] Thomas J, McNaught J, Ananiadou S (2011). Applications of text mining within systematic reviews. Res Syn Meth.

[12] Ananiadou S, Rea B, Okazaki N, Procter R, Thomas J (2009). Supporting Systematic Reviews Using Text Mining. Soc Sci Comput Rev.

[13] Pai M, McCulloch M, Enanoria W, Colford JM (2004). Systematic reviews of diagnostic test evaluations: What's behind the scenes?. ACP J Club.

[14] Higgins JPT, Green S (2008). Cochrane Collaboration. Cochrane handbook for systematic reviews of interventions.

[15] Deeks JJ, Wisniewski S, Davenport C, Deeks J (2013). Guide to the contents of a Cochrane Diagnostic Test Accuracy Protocol. Cochrane Handbook for Systematic Reviews of Diagnostic Test Accuracy.

[16] Leeflang MM, Deeks JJ, Gatsonis C, Bossuyt PM, Cochrane Diagnostic Test Accuracy Working Group (2008). Systematic reviews of diagnostic test accuracy. Ann Intern Med.

[17] Kastner M, Wilczynski NL, McKibbon AK, Garg AX, Haynes RB (2009). Diagnostic test systematic reviews: bibliographic search filters ("Clinical Queries") for diagnostic accuracy studies perform well. J Clin Epidemiol.

[18] Leeflang MM, Scholten RJ, Rutjes AW, Reitsma JB, Bossuyt PM (2006). Use of methodological search filters to identify diagnostic accuracy studies can lead to the omission of relevant studies. J Clin Epidemiol.

[19] Whiting P, Westwood M, Beynon R, Burke M, Sterne JA, Glanville J (2011). Inclusion of methodological filters in searches for diagnostic test accuracy studies misses relevant studies. J Clin Epidemiol.

[20] Kastner M, Haynes RB, Wilczynski NL (2012). Inclusion of methodological filters in searches for diagnostic test accuracy studies misses relevant studies. J Clin Epidemiol.

[21] Wilczynski NL, McKibbon KA, Walter SD, Garg AX, Haynes RB (2013). MEDLINE clinical queries are robust when searching in recent publishing years. J Am Med Inform Assoc.

[22] Ritchie G, Glanville J, Lefebvre C (2007). Do published search filters to identify diagnostic test accuracy studies perform adequately?. Health Info Libr J.

[23] Karimi S, Zobel J, Pohl S, Scholer F (2009). The challenge of high recall in biomedical systematic search.

[24] Cohen AM, Hersh WR, Peterson K, Yen PY (2006). Reducing workload in systematic review preparation using automated citation classification. J Am Med Inform Assoc.

[25] Matwin S, Kouznetsov A, Inkpen D, Frunza O, O'Blenis P (2010). A new algorithm for reducing the workload of experts in performing systematic reviews. J Am Med Inform Assoc.

[26] Cohen AM (2008). Optimizing feature representation for automated systematic review work prioritization. AMIA Annu Symp Proc.

[27] Wallace BC, Trikalinos TA, Lau J, Brodley C, Schmid CH (2010). Semi-automated screening of biomedical citations for systematic reviews. BMC Bioinformatics.

[28] Wallace BC, Small K, Brodley CE, Lau J, Trikalinos TA (2012). Deploying an interactive machine learning system in an evidence-based practice center: abstrackr.

[29] Cohen AM, Ambert K, McDonagh M (2012). Studying the potential impact of automated document classification on scheduling a systematic review update. BMC Med Inform Decis Mak.

[30] Trieschnigg D, Pezik P, Lee V, de Jong F, Kraaij W, Rebholz-Schuhmann D (2009). MeSH Up: effective MeSH text classification for improved document retrieval. Bioinformatics.

[31] Aphinyanaphongs Y, Aliferis CF (2003). Text categorization models for retrieval of high quality articles in internal medicine. AMIA Annu Symp Proc.

[32] Dinnes J, Deeks J, Kirby J, Roderick P (2005). A methodological review of how heterogeneity has been examined in systematic reviews of diagnostic test accuracy. Health Technol Assess.

[33] Lijmer JG, Bossuyt PM, Heisterkamp SH (2002). Exploring sources of heterogeneity in systematic reviews of diagnostic tests. Stat Med.

[34] Riley RD, Dodd SR, Craig JV, Thompson JR, Williamson PR (2008). Meta-analysis of diagnostic test studies using individual patient data and aggregate data. Stat Med.

[35] Ter Riet G, Bachmann LM, Kessels AG, Khan KS (2013). Individual patient data meta-analysis of diagnostic studies: opportunities and challenges. Evid Based Med.

[36] Glanville JM, Lefebvre C, Miles JN, Camosso-Stefinovic J (2006). How to identify randomized controlled trials in MEDLINE: ten years on. J Med Libr Assoc.

